# Isolation in Natural Host Cell Lines of *Wolbachia* Strains *w*Pip from the Mosquito *Culex pipiens* and *w*Pap from the Sand Fly *Phlebotomus papatasi*

**DOI:** 10.3390/insects12100871

**Published:** 2021-09-26

**Authors:** Lesley Bell-Sakyi, Alexandra Beliavskaia, Catherine S. Hartley, Laura Jones, Lisa Luu, Lee R. Haines, James G. C. Hamilton, Alistair C. Darby, Benjamin L. Makepeace

**Affiliations:** 1 Department of Infection Biology and Microbiomes, Institute of Infection, Veterinary and Ecological Sciences, University of Liverpool, Liverpool L3 5RF, UK; alexbel@liverpool.ac.uk (A.B.); Catherine.hartley@liverpool.ac.uk (C.S.H.); lisaluu@liverpool.ac.uk (L.L.); acdarby@liverpool.ac.uk (A.C.D.); blm1@liverpool.ac.uk (B.L.M.); 2 The Pirbright Institute, Pirbright, Surrey GU24 0NF, UK; laura.jones@pirbright.ac.uk; 3 London School of Hygiene and Tropical Medicine, London WC1E 7HT, UK; 4 Vector Biology Department, Liverpool School of Tropical Medicine, Liverpool L3 5QA, UK; lee.haines@lstmed.ac.uk; 5 Division of Biomedical and Life Sciences, Faculty of Health and Medicine, Lancaster University, Lancashire LA1 4YG, UK; j.g.hamilton@lancaster.ac.uk

**Keywords:** mosquito, sand fly, tick cell line, *Wolbachia*, *Culex pipiens*, *Phlebotomus papatasi*, *Lutzomyia longipalpis*, *Culicoides nubeculosus*

## Abstract

**Simple Summary:**

Diverse strains of *Wolbachia* bacteria, carried by many arthropods, as well as some nematodes, interact in many different ways with their hosts. These include male killing, reproductive incompatibility, nutritional supplementation and suppression or enhancement of the transmission of diseases such as dengue and malaria. Consequently, *Wolbachia* have an important role to play in novel strategies to control human and livestock diseases and their vectors. Similarly, cell lines derived from insect hosts of *Wolbachia* constitute valuable research tools in this field. During the generation of novel cell lines from mosquito and sand fly vectors, we isolated two strains of *Wolbachia* and demonstrated their infectivity for cells from a range of other insects and ticks. These new insect cell lines and *Wolbachia* strains will aid in the fight against mosquitoes, sand flies and, potentially, ticks and the diseases that these arthropods transmit to humans and their domestic animals.

**Abstract:**

Endosymbiotic intracellular bacteria of the genus *Wolbachia* are harboured by many species of invertebrates. They display a wide range of developmental, metabolic and nutritional interactions with their hosts and may impact the transmission of arboviruses and protozoan parasites. *Wolbachia* have occasionally been isolated during insect cell line generation. Here, we report the isolation of two strains of *Wolbachia*, *w*Pip and *w*Pap, during cell line generation from their respective hosts, the mosquito *Culex pipiens* and the sand fly *Phlebotomus papatasi*. *w*Pip was pathogenic for both new *C. pipiens* cell lines, CPE/LULS50 and CLP/LULS56, requiring tetracycline treatment to rescue the lines. In contrast, *w*Pap was tolerated by the *P. papatasi* cell line PPL/LULS49, although tetracycline treatment was applied to generate a *Wolbachia*-free subline. Both *Wolbachia* strains were infective for a panel of heterologous insect and tick cell lines, including two novel lines generated from the sand fly *Lutzomyia longipalpis*, LLE/LULS45 and LLL/LULS52. In all cases, *w*Pip was more pathogenic for the host cells than *w*Pap. These newly isolated *Wolbachia* strains, and the novel mosquito and sand fly cell lines reported here, will add to the resources available for research on host–endosymbiont relationships, as well as on *C. pipiens*, *P. papatasi*, *L. longipalpis* and the pathogens that they transmit.

## 1. Introduction

Obligate intracellular bacteria of the genus *Wolbachia* are endosymbionts of a wide range of invertebrate taxa, including many species of arthropod and parasitic nematodes. The nature of the relationships between *Wolbachia* and their hosts cover a wide spectrum, from reproductive manipulations such as cytoplasmic incompatibility (CI) and male killing to nutritional supplementation [1]. CI-inducing strains are of major interest for vector-borne disease control, either through the direct action of CI itself (the “incompatible insect technique”) or via the ability of *Wolbachia* transinfections in mosquitoes to reduce vector competence (population replacement) [1]. *Wolbachia* are currently classified on the basis of gene sequence similarity into ~18 “supergroups” [2].

*Wolbachia* can be cultured in vitro in arthropod cells, facilitating a variety of studies on bacterial–host cell interactions, antibiotic susceptibility and influence on the replication of coinfecting arboviruses or insect-specific viruses [3,4,5,6,7,8,9,10,11,12,13,14,15,16]. Around 16 strains belonging to supergroups A and B have been successfully isolated and grown in insect cell cultures [3,4,5,17,18,19,20,21,22,23]; recently, a novel supergroup F strain from cat fleas was isolated into a tick cell line [24], and a novel supergroup T strain from bedbugs was isolated into a *Drosophila melanogaster* cell line [25]. While the majority of isolations have involved the inoculation of insect-derived material into an established arthropod cell line, in a few cases the *Wolbachia* were cultured directly from the host during primary cell culture initiation [3,21,22]. The outcome of such isolations was either long-lived *Wolbachia*-infected primary cultures [22] or chronically-infected insect cell lines [3,21].

Here, we report the isolation of two strains of *Wolbachia* with contrasting characteristics in naturally-infected host insect cell cultures during cell line generation. A strain of *w*Pip was isolated in both of two new cell lines derived from *Culex pipiens* complex mosquito embryos and larvae, respectively, and a strain of *w*Pap was isolated in a new larva-derived *Phlebotomus papatasi* sand fly cell line. We tested the infectivity of both *Wolbachia* strains for a panel of heterologous insect and tick cell lines, including novel cell lines derived from the sand fly *Lutzomyia longipalpis sensu lato*.

## 2. Materials and Methods

### 2.1. Insects

Eggs from a laboratory colony, established in 2011, of *C. pipiens* complex mosquitoes (Caldbeck line, mixed *Culex pipiens pipiens* and *Culex pipiens molestus* originating from Surrey, UK [26]) were originally developed and supplied by The Pirbright Institute under BBSRC project code BBS/E/I/00007039. Eggs from a colony of *P. papatasi* sand flies (strain Turkey (PPTK) originally supplied by the Walter Reed Army Institute of Research for distribution by BEI Resources, NIAID, NIH, Manassas, VA, USA) were provided by the Liverpool School of Tropical Medicine. Eggs from laboratory colonies of the Jacobina (3-methyl-α-himachalene male pheromone type) and Sobral (sobralene male pheromone type) strains of the sand fly *L. longipalpis* species complex, originating from Brazil [27,28], were provided by Lancaster University.

### 2.2. Initiation of C. pipiens and P. papatasi Primary Cell Cultures

**Figure 1:**
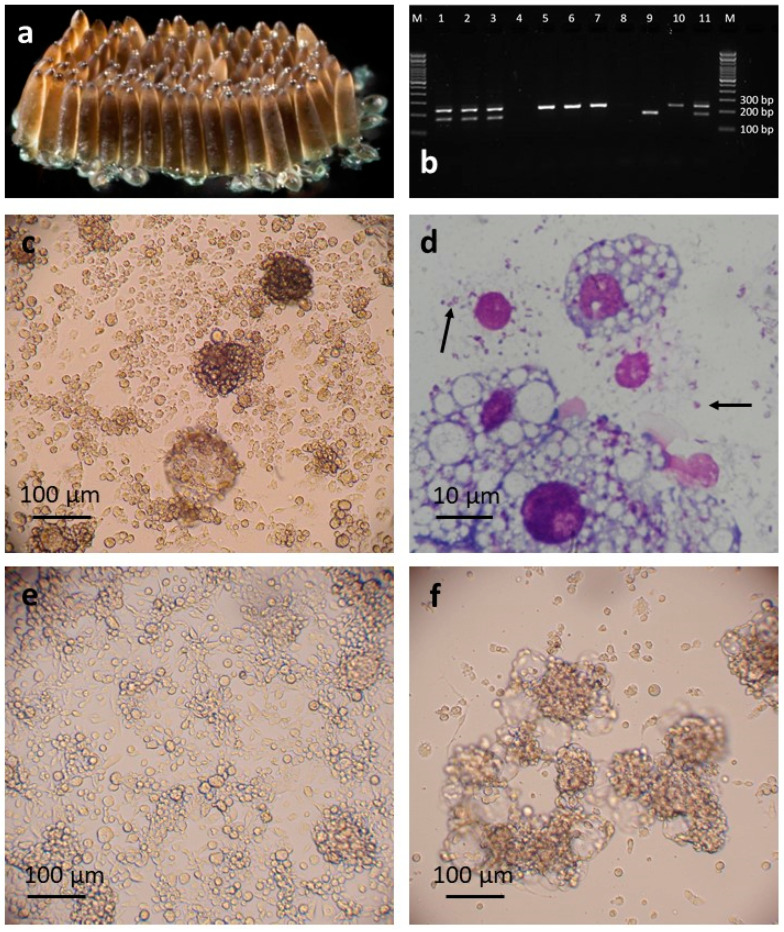
*Culex pipiens* cell lines CPE/LULS50 and CPL/LULS56. (**a**) *C. pipiens* eggs. (**b**) Agarose gel electrophoresis of CQ11 microsatellite locus PCR products obtained from DNA extracted from CPE/LULS50 cells (lanes 1–3), CPL/LULS56 (lanes 5–7), *Culex pipiens pipiens* mosquito (lane 9), *Culex pipiens molestus* mosquito (lane 10), *C. p. pipiens* and *C. p. molestus* hybrid (lane 11); negative controls are in lanes 4 and 8. (**c**) CPE/LULS50 cells at passage 1, 6 months after initiation, live, inverted microscope. (**d**) CPE/LULS50 cells showing *Wolbachia* bacteria inside cells and extracellularly (arrows); Giemsa-stained cytocentrifuge smear. (**e**) CPE/LULS50 cells at passage 18, 17 months after initiation; live, inverted microscope. (**f**) CPL/LULS56 cells at passage 5, 17 months after initiation; live, inverted microscope.

*C. pipiens* eggs (Figure 1a) laid within the previous 48 h were surface-sterilised by immersion in 0.1% benzalkonium chloride for 10 min and 70% ethanol for 1 min, followed by two rinses in Hank’s balanced salt solution (HBSS). Any hatched larvae were removed, unhatched eggs were crushed in 0.5-mL HBSS in a plastic petri dish using the flattened end of a glass rod, the resultant tissue suspension was diluted with ~2-mL HBSS and centrifuged at 200× *g* for 5 min, and the tissue pellet was resuspended in 0.5-mL complete culture medium and placed in the bottom of a flat-sided culture tube (Nunc, Thermo Fisher, Loughborough, UK). The culture medium comprised L-15 (Leibovitz) medium supplemented with 10% tryptose phosphate broth (TPB), 20% foetal bovine serum (FBS), 2mM L-glutamine (L-glut), 100 units/ mL penicillin, 100 µg/ mL streptomycin (pen/strep) and 50 µg/ mL amphotericin B (L-15; all ingredients were obtained from Invitrogen, Thermo Fisher, Loughborough, UK or from Sigma-Aldrich, Gillingham, UK). The sealed culture tube was incubated in ambient air in a dry incubator at 28 °C. The medium was changed weekly by the removal and replacement of 1/2–2/3 of the volume; amphotericin B was omitted after the first medium change, and the total volume was gradually increased to 2 mL.

A second *C. pipiens* egg batch was processed in the same way, but not all the eggs were crushed, and larvae emerged within the first 24 h in vitro. On the day after initiation, 7 larvae were removed from the culture, chopped into several pieces in a drop of L-15 using a scalpel and returned to the tube with an additional 0.5 mL of medium. On the following day, the contents of the tube were centrifuged at 200× *g* for 5 min, the supernate was saved and the pellet resuspended in 0.5 mL of trypsin solution (500 µg/ mL in PBS). The larval pieces were chopped into smaller fragments and incubated with an additional 0.5 mL trypsin solution for 10 min at 37 °C; 0.5 mL of the saved supernate was added, and the tissue suspension was centrifuged at 400× *g* for 5 min. The tissue pellet was resuspended in the remaining 0.8 mL saved medium and returned to the tube, which was incubated at an angle of ~10°. Thereafter, the medium was changed weekly, gradually increasing the total volume to 2 mL.

**Figure 2:**
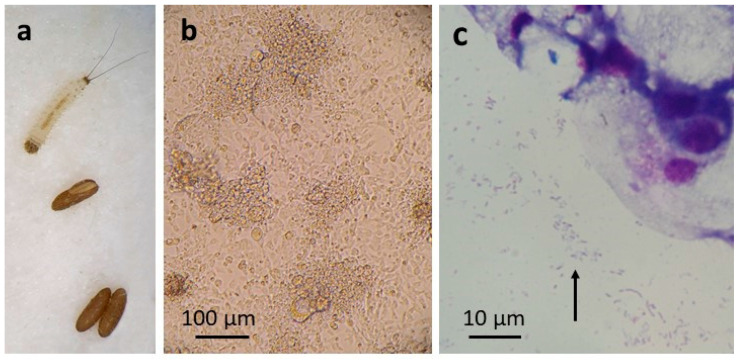
*Phlebotomus papatasi* cell line PPL/LULS49. (**a**) *P. papatasi* eggs (bottom), empty eggshell (centre) and first instar larva (top). (**b**) PPL/LULS49 cells at passage 9, 15 months after initiation; live, inverted microscope. (**c**) PPL/LULS49 cells showing extracellular *Wolbachia* (arrow); Giemsa-stained cytocentrifuge smear.

*P. papatasi* eggs (Figure 2a) laid approximately 5–8 days previously were surface-sterilised by immersion in 0.1% benzalkonium chloride for 8 min and 70% ethanol for 5 min, followed by two rinses in HBSS. The eggs were then crushed as above in a mixture of 0.5 mL HBSS and 1 mL complete culture medium comprising L-15B medium [29] supplemented with 10% TPB, 10% FCS, 0.1% bovine lipoprotein concentrate (MP Biomedicals, Thermo Fisher, Loughborough, UK), L-glut, pen/strep, 50 µg/mL amphotericin B and 5 µg/mL tetracycline hydrochloride (L-15B). The resultant mixture of embryonic tissues, eggshells and uncrushed eggs was allowed to stand for 2 min; the supernate was then transferred to a flat-sided culture tube, and the pellet was submitted to a second round of crushing in L-15B and sedimentation. The resultant supernate was transferred to a second culture tube, and the pellet of eggshells and uncrushed eggs were placed in a third tube. All the tubes were incubated at 28 °C. The medium was changed as above after a week; at which point, the hatched larvae in the third tube were transferred to a new tube in L-15B. Amphotericin B and tetracycline were omitted from the subsequent weekly medium changes. Twenty-five days after initiation, the first two tubes were discarded, as they did not contain any viable tissues or cells, and 28 hatched larvae were removed from the third tube, chopped into at least three pieces each in HBSS using watchmakers’ forceps and centrifuged at 400× *g* for 5 min. The supernate was discarded, and the tissue pellet was resuspended in 0.5 mL of trypsin solution and incubated for 5 min at 37 °C. An equal volume of complete culture medium comprising HBSS supplemented with 0.5% lactalbumin hydrolysate (Sigma-Aldrich, Gillingham, UK), 20% FBS, L-glut and pen/strep (H-Lac) was added, the tissue suspension was centrifuged at 400× *g* for 5 min and the resultant pellet was resuspended in 1 ml H-Lac and placed in a new culture tube at 28 °C. Thereafter, this culture was maintained in a 1:1 mixture of L-15B and H-Lac media.

### 2.3. C. pipiens and P. papatasi Cell Line Generation

The primary cultures were monitored by weekly inverted microscope examination. When clumps of proliferating cells appeared, the cultures were reseeded by vigorous pipetting to encourage cell growth. When cells were clearly growing, and the pH of the culture medium became acidic (orange or yellow colour) indicating a high level of active metabolism, an equal volume of fresh medium was added to the culture, the cells and tissue clumps were resuspended by vigorous pipetting and half of the resultant suspension was transferred to a new flat-sided culture tube. After several subcultures, cells were passaged into sealed, 25-cm^2^ culture flasks. Cells were cryopreserved in medium with 20% FBS and 10% DMSO and resuscitated as described previously for *Culicoides nubeculosus* cell lines [30]. Primary *C. pipiens* and *P. papatasi* cultures and young cell lines were periodically examined by preparation of Giemsa-stained cytocentrifuge smears as described previously [30]. Absence of contaminating *Mycoplasma* in all young cell lines was confirmed using two commercial tests, the Mycoalert Mycoplasma Detection Kit (Lonza, Fisher Scientific, Loughborough, UK) and the PCR Mycoplasma Test Kit (Promocell, VWR, Lutterworth, UK), following the manufacturers’ instructions.

### 2.4. Confirmation of Species Origin and Identification of Wolbachia

DNA was extracted from resuspended *C. pipiens* and *P. papatasi* cells using a DNeasy Blood and Tissue kit (Qiagen, Hilden, Germany), following the manufacturer’s instructions for cultured cells. To confirm the species origin, a 696-bp fragment of the mitochondrial *cytochrome oxidase 1* (*cox1*) gene was amplified by PCR (forward primer: 5′-GGATTTGGAAATTGATTAGTTCCTT-3′ and reverse primer: 5′-AAAAATTTTAATTCCAGTTGGAACAGC-3′) from the mosquito cells as described previously [31], and an 851-bp fragment of the eukaryotic 18S rRNA gene was amplified by PCR (forward primer EukA: 5′-AACCTGGTTGATCCTGCCAGT-3′ and reverse primer EukB: 5′-TGATCCTTCTGCAGGTTCACCTAC-3′) from the sand fly cells as described previously [32].

To determine the ecotype of the *C. pipiens* cells, conventional PCR targeting the CQ11 microsatellite locus was carried out, based on a method and the primers described previously [33], on DNA from cultured cells with DNA extracted from *C. p. pipiens*, *C. p. molestus* and hybrid mosquitoes as the positive controls. The PCR reactions consisted of 1 × PCR buffer, 1.5 mM MgCl_2_, 0.2 mM dNTP mix, 0.16-µg/µL bovine serum albumin, 1 U Platinum™ Taq DNA polymerase (Invitrogen, Thermo Fisher, Loughborough, UK), 0.24-µM forward primer CQ11F, 0.32 µM reverse primer PipCQ11R, 0.14 µM reverse primer MolCQ11R and 3 µL DNA, made up to 25 µL with nuclease free water. The thermal cycling of the reaction consisted of 94 °C for 2 min, followed by 35 cycles of 94 °C for 30 s, 55 °C for 30 s and 72 °C for 1 min and a final elongation step of 72 °C for 10 min. The PCR products were visualised by agarose gel electrophoresis.

To detect and identify *Wolbachia*, DNA extracted from cultured cells was subjected to conventional PCRs amplifying fragments of the pan-bacterial 16S rRNA gene and the *Wolbachia wsp* gene following the respective published protocols [34,35]. The PCR products were visualised by agarose gel electrophoresis. Positive PCR products were purified using a PureLink Quick Gel Extraction and PCR Purification Combo kit (Thermo Fisher Loughborough, UK) following the manufacturer’s instructions, and Sanger-sequenced in both directions (Eurofins Genomics, Ebersberg, Germany). The sequences were analysed in Bioedit v.7.2.5 [36].

Phylogenetic analysis of the *Wolbachia* sequences was carried out using sequences from the 16S rRNA and *wsp* genes. Initial multiple alignments for the 16S rRNA and *wsp* genes were constructed using MAFFT v7.480 [37] with the L-INS-I algorithm. The alignments were further analysed using BMGE v1.12 [38] to select those that were suitable for phylogenetic inference. The alignments were then concatenated into a single alignment for further analysis. To select the model that best fitted our data, modeltest-ng [39] was run on the concatenated alignment. The best model was GTR + I+G4 according to the AIC and AICc tests, and TPM2uf + I+G4 according to the BIC criteria. The first model was chosen, because it showed the faster bootstrap convergence. Tree reconstruction was carried out using RAxML-NG v. 1.0.2 generating 1000 bootstraps [40]. The resulting phylogenetic tree was visualised using Interactive Tree of Life v4 [41].

### 2.5. Heterologous Tick and Insect Cell Lines

The established tick cell lines BME/CTVM23 [42], ISE6 [43] and IRE/CTVM20 [44] and insect cell lines Sf9 (moth) [45,46], C6/36 (mosquito) [47] and CNE/LULS44 (biting midge) [30] were maintained at 28 °C, as shown in Table 1. Two new insect cell lines were included in the study (Table 1). The *Lutzomyia longipalpis* cell line LLE/LULS45 was derived from embryos of the Jacobina strain following the same procedure for processing eggs as described above for *P. papatasi*, which yielded a culture of viable embryonic cells and tissues that gave rise to the cell line. The *L. longipalpis* cell line LLL/LULS52 was derived from eight larvae of the Sobral strain, 13 days after culture initiation, following the same procedure as described above for *P. papatasi* larvae derived from processed and cultured unhatched eggs. Establishment of the cell lines LLE/LULS45 and LLL/LULS52 was achieved with successful cryopreservation at 12 (passage 6) and 11 (passage 4) months, respectively. The origins of both cell lines were confirmed as the *L. longipalpis* species complex by sequencing *cox1* PCR products amplified using the primers LCO1490 and HCO2198, as described previously [48]. A 601-bp sequence obtained from LLE/LULS45 was 99.17% identical to a sequence from *Lutzomyia cruzi* (a member of the *L. longipalpis* species complex) and 99.00% identical to a sequence from *L. longipalpis* (query cover 100%, GenBank accession numbers KP112575.1 and KP112586.1), both from Mato Grosso State, Brazil [49]. A 651-bp sequence obtained from LLL/LULS52 was 99.54% identical to a sequence from another member of the *L. longipalpis* species complex, *Lutzomyia alencari*, and 99.08% identical to a sequence from *L. longipalpis* (query cover 100%, GenBank accession numbers KP112569.1 and KP112590.1), both from Espirito Santo, Brazil [49]. To date, LLE/LULS45 and LLL/LULS52 have been taken through 22 and 19 passages, respectively, at 28 °C. All heterologous arthropod cell lines used in the present study were confirmed to be free of *Mycoplasma* using two commercial kits, as described in Section 2.3.

### 2.6. Inoculation of Heterologous Cell Lines

Supernates removed from *Wolbachia*-infected *C. pipiens* and *P. papatasi* cultures were centrifuged at 2000× *g* for 5 min to remove any intact cells; the absence of contaminating host cells was confirmed by examination of Giemsa-stained cytocentrifuge smears, and 0.3–0.5 mL aliquots were added to cultures of heterologous tick and insect cell lines (Table 1) in sealed, flat-sided tubes. Inoculated cultures were maintained at 28 °C with weekly medium changes and were monitored by the periodic preparation of Giemsa-stained cytocentrifuge smears. When intracellular bacteria were visible, the presence of *Wolbachia* was confirmed by DNA extraction and subjecting the DNA to quantitative PCR (qPCR) targeting the *Wolbachia* 16S rRNA gene as described previously [50], except that the reaction mix comprised Sensimix No. Rox SYBR (Bioline/SLS, Nottingham, UK). If the cells became heavily infected, with at least 50% of the cells containing bacteria and/or the appearance of a cytopathic effect (CPE), infected cultures were passaged by transfer of 0.2 mL of cell suspension to a fresh tube of the same cell line. If the infected cultures did not display CPE, they were passaged by resuspending the cells and transferring half of the cell suspension into a new flat-sided tube. Infected cultures were cryopreserved with 10% DMSO in vapour-phase liquid nitrogen, as described previously [51].

**Table 1:** Origins of, and culture media used for the propagation of, arthropod cell lines tested for the ability to support growth of *Wolbachia* strains *w*Pip and *w*Pap.

Cell Line	Arthropod Species	Culture Medium	Passage Level	Reference
BME/CTVM23	*Rhipicephalus microplus*	L-15	~85	[42]
ISE6	*Ixodes scapularis*	L-15B300 ^1^	~83	[43]
IRE/CTVM20	*Ixodes ricinus*	L-15/L-15B ^2^	~186	[44]
Sf9	*Spodoptera frugiperda*	TC100 ^3^	? ^4^	[45,46]
C6/36	*Aedes albopictus*	L-15	? ^4^	[47]
CNE/LULS44	*Culicoides nubeculosus*	L-15	~15	[29]
LLE/LULS45	*Lutzomyia longipalpis*	L-15B	~2	This study
LLL/LULS52	*Lutzomyia longipalpis*	L-15/L-15B ^2^	~5	This study

^1^ As L-15B but with three parts basal L-15B and one part ultrapure water [52]. ^2^ Equal parts of complete L-15 and L-15B media. ^3^ TC100 medium (Invitrogen) supplemented with 10% FBS, L-glut and pen/strep. ^4^ Passage level unknown.

In a preliminary experiment, the tick cell line IRE/CTVM20, selected because it comprises cells easily resuspended by pipetting, was used to assess the effect of infection with *w*Pap on growth rate. Uninfected cells, and cells infected with *w*Pap at passage 1, were seeded in 2 mL of medium in three replicate flat-sided tubes at a density of 1.41 × 10^6^ cells/mL and incubated at 28 °C. The medium was changed on days 7 and 13 by removal and replacement of 1.5 mL. The cells in each tube were resuspended and counted using a haemocytometer on days 6, 10 and 14. Two-way ANOVA comparing the cell counts was performed on GraphPad Prism version 9.2.0 for Windows (GraphPad Software, San Diego, CA, USA) as a main effects-only model.

### 2.7. Removal of Wolbachia from Infected C. pipiens and P. papatasi Cell Lines

*Wolbachia*-infected *C. pipiens* and *P. papatasi* cultures were treated weekly with tetracycline hydrochloride (Sigma-Aldrich, Gillingham, UK) added to the culture supernate following a medium change to give a final concentration of 5 µg/mL, over a period of at least 12 weeks. Absence of *Wolbachia* was determined by extracting DNA as described above from treated cells at least 1 week after the cessation of tetracycline treatment, and by subjecting the DNA to qPCR targeting the *Wolbachia* 16S rRNA gene as described above.

## 3. Results

### 3.1. C. pipiens Cell Lines

A single primary culture in L-15 was obtained from embryonic tissues released from *C. pipiens* eggs. Patches of twitching tissues resembling muscle fibres were visible after 8 days in culture, and small patches of growing round cells were seen after three weeks. These increased rapidly in number, and the first passage from the primary culture was carried out at 9 weeks. The early subcultures comprised attached round cells of varying size and granularity, sometimes forming clumps and accompanied by floating multicellular vesicles (Figure 1c). The cells were successfully cryopreserved and resuscitated at passage 3, 23 weeks after initiation; at this point, the cell line was considered established and was designated CPE/LULS50. DNA was then extracted to confirm the species origin and to screen for any contaminating bacteria. A PCR targeting the *cox1* gene confirmed that the CPE/LULS50 cells belonged to the *C. pipiens* complex (100% identity with 99% query coverage to a sequence from a *C. pipiens* mosquito from Greece, GenBank accession number MN850560.1); a further ecotype analysis revealed that the cell line contained a mixture of *C. p. pipiens* and *C. p. molestus* cells and possibly cells derived from hybrids of the two ecotypes (Figure 1b).

A pan-bacterial PCR targeting the 16S rRNA gene revealed that the CPE/LULS50 cells were infected with a *Wolbachia* with 100% similarity to the Pel sub-strain of *w*Pip [53] (100% query cover, GenBank accession number AM999887.1). The sequence chromatogram showed individual, evenly-spaced peaks at the nucleotide bases with no overlapping signals, indicating good sequencing quality and suggesting absence of any other contaminating bacteria. The presence of intracellular *Wolbachia*-like bacteria in the CPE/LULS50 cells was confirmed by examination of Giemsa-stained cytocentrifuge smears (Figure 1d). At this point, 28 weeks after initiation, treatment of some cultures with tetracycline was commenced with the aim of obtaining a *Wolbachia*-free subline. Four weeks later, the untreated cells began to die, presumably as a result of the *Wolbachi*a infection, as no other bacterial contaminants were detected by microscopy, pan-bacterial 16S rRNA PCR or *Mycoplasma* screening, and all the surviving CPE/LULS50 cultures, including the parental primary culture, were treated with tetracycline for at least 12 weeks. Samples screened by qPCR at 1, 11 18 and 35 weeks (0–10 passages) following the cessation of tetracycline treatment were negative for *Wolbachia*. *Wolbachia*-free CPE/LULS50 cells were cryopreserved at passage 12, and, at the time of writing, the cell line has reached passage 25. CPE/LULS50 comprises predominantly attached, round and spindle-shaped cells of varying sizes and granularities that form occasional three-dimensional clumps (Figure 1e)

A second *C. pipiens* primary culture was obtained from seven macerated larvae that hatched from uncrushed eggs from a separate egg batch. The cell growth was much slower than in the primary culture that gave rise to CPE/LULS50, and the first subculture was carried out at 27 weeks. This culture comprised mainly floating multicellular vesicles, with only small numbers of attached round cells. At 35 weeks, a Giemsa-stained cytocentrifuge smear revealed a heavy infection with an intracellular bacterium, confirmed by PCR to be the *w*Pip strain of *Wolbachia*, and the tetracycline treatment of this culture series was initiated, leaving the parent primary culture untreated. Samples of treated cells screened by qPCR at 18 and 35 weeks following the cessation of tetracycline treatment were negative for *Wolbachia*. Cell growth continued slowly until 14 months after the primary culture initiation, when more rapid growth commenced. Cells of this line, designated CPL/LULS56, were successfully cryopreserved at passage 3, 16 months after initiation; at this point, the cell line was considered established. At the time of writing, CPL/LULS56 cells had reached passage 11; they still comprised predominantly floating clumps of rounded cells in association with multicellular vesicles, but the proportion of attached round and spindle-shaped cells was slowly increasing (Figure 1f). The *cox1* PCR confirmed that CPL/LULS56 was derived from *C. pipiens* complex mosquitoes, and ecotyping revealed that, in contrast to the embryo-derived line CPE/LULS50, the larva-derived line is derived only from *C. p. molestus* (Figure 1b).

### 3.2. P. papatasi Cell Line

The single *P. papatasi* primary larva-derived culture in H-Lac/L-15B medium was maintained with a gradually increasing medium volume; seven weeks after the larvae were macerated, several small patches of growing cells were seen. These gradually increased in size over the subsequent five months; by which time, the culture contained many cells of different sizes forming patches, clumps and multicellular vesicles (Figure 2b). The first subculture was carried out at 28 weeks, and cells at passage 5 were successfully cryopreserved and resuscitated when the cell line, designated PPL/LULS49, was 13 months old and considered established. Sequencing of the eukaryotic 18S rRNA PCR product confirmed the species origin as *P. papatasi* (100% identity with 100% query cover to a sequence from a *P. papatasi* sand fly from Cyprus [54], GenBank accession number AJ244409.1).

When the PPL/LULS49 cells were nearly a year old, it became evident that bacteria were growing in the cultures, manifest as very small, faintly stained, rod-shaped and pleomorphic organisms both intra- and extracellular (Figure 2c). Pan-bacterial 16S rRNA PCR screening revealed them to be a *Wolbachia*, with highest similarity (99.69%, 100% query cover) to *Wolbachia* endosymbionts of the spittlebug *Cosmoscarta heros*, the weevil *Curculio okumai* and the ant *Anoplolepis gracilipes* (GenBank accession no. AB772264.1, AB746402.1 and GQ275135.1, respectively). As with the *Wolbachia* detected in *C. pipiens* cells, examination of the sequence chromatogram indicated good sequencing quality and suggested absence of any other contaminating bacteria; screening of the PPL/LULS49 cells for *Mycoplasma* gave negative results. A *Wolbachia*-specific PCR targeting the *wsp* gene yielded a 316-bp sequence, confirming that the PPL/LULS49 cells harboured *w*Pap (100% identity, 100% query cover, with the *Wolbachia* strain *w*Pap detected in *P. papatasi* from India, GenBank accession number AF237882.1 [55]). In contrast to *w*Pip in the *C. pipiens* cells, *w*Pap was often seen extracellularly and did not appear to be pathogenic for the *P. papatasi* cells. However, tetracycline treatment was commenced, and eventually, after an initial period when the cells appeared to be adapting to the loss of the bacteria with suboptimal growth, a healthy subline was obtained. Cultures of this subline were screened by qPCR 1, 8 and 25 weeks (0–4 passages) after the cessation of the tetracycline treatment and found to be free of *Wolbachia*. The original line of *Wolbachia*-infected cells, henceforward redesignated as PPL/LULS49/*w*Pap, was maintained in parallel. At the time of writing, PPL/LULS49/*w*Pap had reached passage 23, while the *Wolbachia*-free subline of PPL/LULS49 had reached passage 15; both sublines comprise sheets and clumps of attached cells composed of a mixture of small, rounded cells, some fibroblast-like and epithelial-like cells and floating, multicellular vesicles.

### 3.3. Phylogeny of wPip and wPap

**Figure 3:**
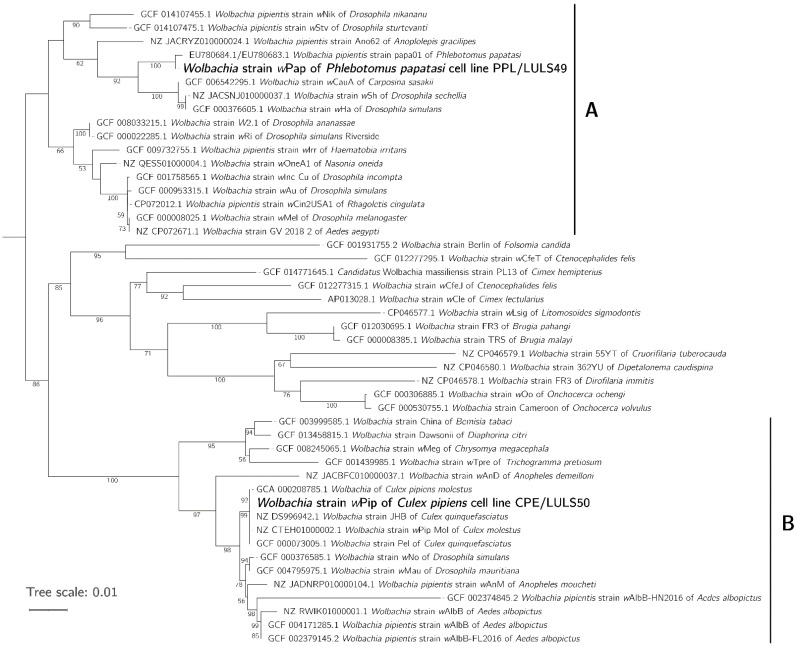
Phylogenetic analysis of the *Wolbachia* strains *w*Pip and *w*Pap isolated in cell lines CPE/LULS50 and PPL/LULS49, respectively. Concatenated phylogenetic tree generated from 16S rRNA and *wsp* sequences obtained in the present study (in **bold**) and previously published in GenBank, showing the accession numbers and host species. Supergroups **A** and **B** are indicated by capital letters to the right of the tree.

The resultant 16S rRNA and *wsp* gene sequences from the novel *w*Pip and *w*Pap isolates were compared with published sequences to determine their relationship with other *Wolbachia* strains. A concatenated tree prepared using sequences from both genes placed our *w*Pip strain in supergroup B, together with other *Wolbachia* strains isolated from *C. pipiens* complex mosquitoes, and close to strains from *Aedes* and *Anopheles* spp. mosquitoes and *Drosophila* spp. fruit flies (Figure 3). Our *w*Pap strain was placed in supergroup A, clustering with another *Wolbachia* from *P. papatasi* and close to strains from *Drosophila* spp. fruit flies and the peach fruit moth *Carposina sasakii* (Figure 3).

The novel gene sequences generated in this study were deposited in GenBank under the following accession numbers: MZ577349 (CPE/LULS50 *cox1*), MZ577351(PPL/LULS49 18S rRNA), MZ577353 (LLE/LULS45 *cox1*), MZ577352 (LLL/LULS52 *cox1*), MZ577347 (*w*Pip 16S rRNA), MZ577346 (*w*Pip *wsp*), MZ577348 (*w*Pap 16S rRNA) and MZ577354 (*w*Pap *wsp*).

### 3.4. Infectivity of wPip and wPap for Heterologous Cell Lines

**Figure 4:**
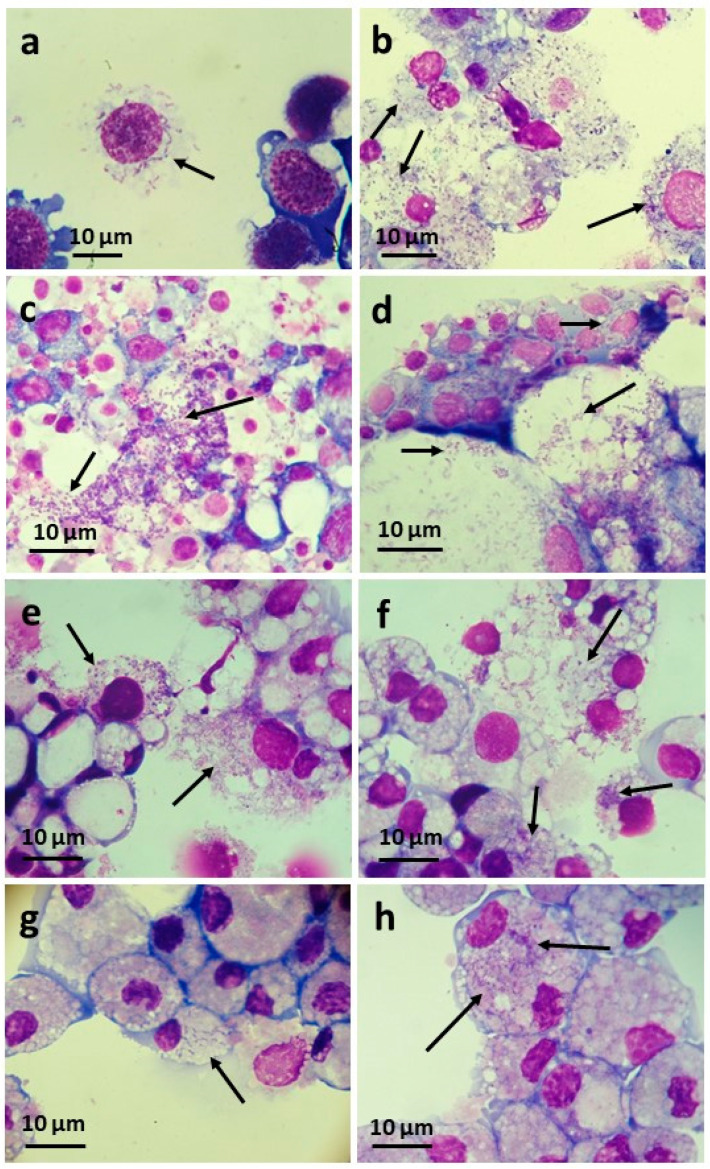
*Wolbachia* strains *w*Pip and *w*Pap (arrows) in Giemsa-stained cytocentrifuge smears of heterologous cell lines. (**a**) *w*Pip in Sf9 cells. (**b**) *w*Pip-infected C6/36 cells at passage 4. (**c**) *w*Pip at passage 3 in CNE/LULS44 cells. (**d**) *w*Pip at passage 2 in LLE/LULS45 cells. (**e**) *w*Pip in BME/CTVM23 cells. (**f**) *w*Pap in BME/CTVM23 cells. (**g**) *w*Pip in IRE/CTVM20 cells. (**h**) *w*Pap in IRE/CTVM20 cells.

Cell-free CPE/LULS50 supernate containing *w*Pip bacteria was inoculated into the insect cell lines Sf9 and C6/36 and the tick cell lines BME/CTVM23 and ISE6 (Table 2). All recipient cell lines became visibly infected with *Wolbachia*, as determined by the examination of Giemsa-stained smears between one (tick cells and Sf9) and nine (C6/36) weeks later (Figure 4a,b). The *w*Pip infection was taken through five passages onto naïve BME/CTVM23 cells over a 4-month period before being cryopreserved. The infection in the two tick cell lines was pathogenic, causing heavy infections within two weeks and cell death after 6 weeks. In contrast, the *w*Pip infection was tolerated better by the insect cell lines, with reduced growth rate and metabolism (manifest as higher pH in infected compared to uninfected cultures) but prolonged survival of the culture as a whole. The infection was slow to develop in C6/36 cells, but the infected cells could be split 1:1 successfully and were taken through eight passages over a 9.5-month period before being cryopreserved.

Cell-free CPL/LULS56 supernate containing *w*Pip bacteria was inoculated into the tick cell lines BME/CTVM23 and IRE/CTVM20 and the insect cell lines CNE/LULS44, LLE/LULS45 and LLL/LULS52 (Table 2). All recipient cell lines became visibly infected with *Wolbachia* between two (tick cells) and three (insect cells) weeks later; development of CPE ensued rapidly in the BME/CTVM23 cells (Figure 4e), causing death of the culture at 7 weeks, and more slowly in the IRE/CTVM20 (Figure 4g) and insect cells, causing death of the cultures between 9 (tick) and 14 (insect) weeks later. *w*Pip was taken through three passages onto naïve CNE/LULS44 (Figure 4c) and two passages onto naïve LLE/LULS45 (Figure 4d) cells over an 8-month period.

**Figure 5:**
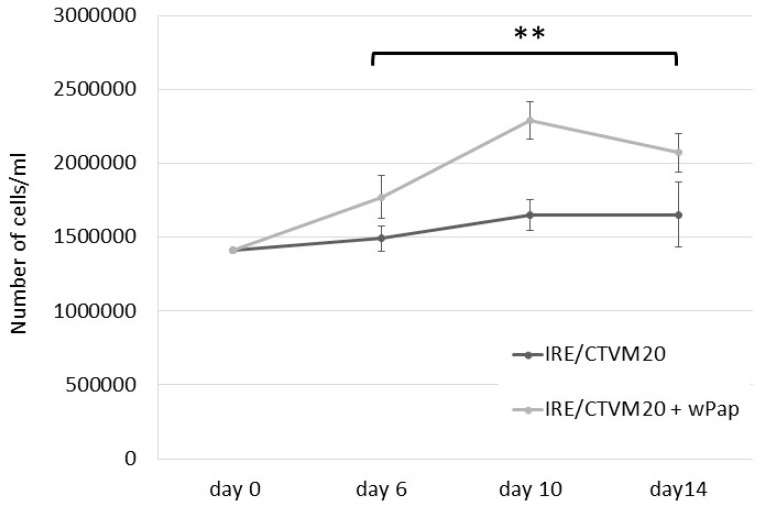
Effect of *Wolbachia* strain *w*Pap infection on the growth rate of IRE/CTVM20 cells. Uninfected and wPap-infected IRE/CTVM20 cells at passage 1 were seeded in flat-sided tubes in triplicate, and the cells were counted using a haemocytometer on days 6, 10 and 14. The graph shows the means of three cell counts with standard errors. ** Indicates significant differences between the groups (two-way ANOVA, *p* = 0.0012).

Cell-free PPL/LULS49 supernate containing *w*Pap bacteria was inoculated into the three tick cell lines BME/CTVM23, ISE6 and IRE/CTVM20 and four insect cell lines, Sf9, CNE/LULS44, LLE/LULS45 and LLL/LULS52 (Table 2). Infection was detectably established between one and six weeks later in the tick cell lines (Figure 4f,h) and all heterologous insect lines except CNE/LULS44, which was shown to be *Wolbachia*-negative by qPCR 15 weeks after the inoculation, while LLE/LULS45 cells inoculated at the same time were heavily infected. *w*Pap was taken through three passages onto naïve BME/CTVM23 cells over 3.5 months. Compared to *w*Pip, *w*Pap was less pathogenic for the tick and insect cell lines and did not always result in death of the infected cultures; it was possible to subculture the *w*Pap-infected *L. longipalpis* and IRE/CTVM20 cells at least twice for each cell line. In a preliminary experiment, IRE/CTVM20 cells infected with *w*Pap grew to a significantly higher density (*p* = 0.0012, two-way ANOVA) than uninfected cells over a 14-day period (Figure 5).

## 4. Discussion

In the present study, we report two new cell lines derived from *C. p. pipiens*/*C. p. molestus* complex mosquitoes, one new cell line derived from *P. papatasi* sand flies and two new cell lines derived from *L. longipalpis* sand flies. While cell lines have previously been reported from all three species [56,57,58,59,60,61], these were derived from embryos or adult ovaries, and, at the time of writing, none were available from major international culture collections, apart from the *L. longipalpis* cell line LLE/LULS40 [61]. As far as we know, PPL/LULS49, LLL/LULS52 and CPL/LULS56 are the first larva-derived cell lines generated from *P. papatasi*, *L. longipalpis* and *C. p. molestus* respectively. According to the Cellosaurus website [62,63], a cell line derived from the *Culex pipiens pallens* first instar larvae has been reported [64], but we were unable to access the paper online to confirm the method used. Larva-derived mosquito cell lines, including the *Aedes albopictus* line from which C6/36 cells were derived [47], were first reported by Singh in 1967 [65], although their approach was slightly different to ours as they macerated the *Aedes* spp. mosquito larvae immediately after hatching from surface-sterilised eggs. In our case, the intact mosquito or sand fly larvae were maintained in culture for 1–25 days prior to maceration, indicating that a degree of flexibility in timing can be used with this approach to cell line generation from larval insects. Moreover, together with LLE/LULS40, the two new *L. longipalpis* lines LLE/LULS45 and LLL/LULS52 form a set of cell lines derived from sand fly strains with three different, well-defined pheromone profiles [27,28,66] that will, when sequenced, help to reveal the genetics underlying these characteristics.

As part of the process of establishing the cell lines, we used order-specific PCR assays and amplicon sequencing to confirm the species origin of the cells, especially important for studies involving arthropod–symbiont relationships. For all the novel cell lines reported here, the species origin was confirmed, and the CPE/LULS50 cell line was found to contain cells derived from both *C. p. pipiens* and *C. p. molestus*, reflecting the composition of the parent mosquito colony. Interestingly, the CPL/LULS56 cell line, which was derived from far fewer individuals (*n* = 7) of the same colony than CPE/LULS50 (*n* = ~100), was found to comprise only *C. p. molestus* cells. There have been several previous reports of the misidentification of arthropod cell lines, in most cases those established prior to the general availability of molecular screening techniques. The RML-12 cell line, originally described as *Aedes aegypti* [5] and used to propagate several *Wolbachia* strains [5,10,23], was later found to be derived from *Ae. albopictus* [67]. Similarly, the RML-15 cell line, originally reported as derived from the tick *Dermacentor variabilis* [68], was later found to be derived from *Rhipicephalus sanguineus* ticks [69]. Most alarmingly, the first cell line reported from a crustacean, the crayfish *Orconectes limosus* [70], was later found to be an amoeba [71].

Although the main aim of setting up primary arthropod cell cultures has usually been to generate cell lines, the coincidental isolation of arthropod-borne bacteria has often been an unexpected but beneficial by-product. In addition to generating novel cell lines from *C. pipiens* complex mosquitoes and *P. papatasi* sand flies, we isolated two strains of *Wolbachia* with contrasting characteristics. Although the *w*Pip strain was initially tolerated by both *C. pipiens* primary cultures and early subcultures derived from them, it began to cause CPE after 8 months in vitro, and it was necessary to treat all surviving cultures (apart from the parent culture of CPL/LULS56, which still survived with a chronic *w*Pip infection at the time of writing, 19 months post-initiation) with tetracycline to save the cell lines. In contrast, *w*Pap did not become manifest until the *P. papatasi* cells had grown for a year and was tolerated with little or no CPE to at least passage 22 (27 months post-initiation). Tetracycline treatment resulted in a *Wolbachia*-free subline, currently maintained alongside the original PPL/LULS49/*w*Pap cell line. This difference in pathogenicity between the two *Wolbachia* strains was also seen in heterologous cell lines, with *w*Pip causing rapid CPE in tick, midge and sand fly cell lines, while CPE caused by *w*Pap took longer to develop in two of the tick cell lines and was negligible in the third tick cell line and both sand fly cell lines, allowing the infected cells to be subcultured.

While a strain of *w*Pip was previously isolated into an *Ae. albopictus* cell line from *C. pipiens* eggs of unspecified geographical origin [4], the present study is the first report of the isolation of *w*Pip in *C. pipiens* cell cultures. The *C. pipiens* complex is infected ubiquitously with *w*Pip worldwide, and the symbiont is divided into five clades (*w*PipI–*w*PipV), generating the most complex pattern of CI recognised in nature to date [72]. Further characterisation is required to determine to which clade the *w*Pip strain isolated in the present study belongs. Interestingly, a recent report demonstrated that *w*Pip is unusual in failing to impede dengue virus dissemination when transinfected into *Ae. aegypti*, providing opportunities to unravel the mechanisms of viral inhibition [73]. The present study also represents the first report of isolation and in vitro propagation of any strain of *w*Pap, which is a common symbiont of wild *P. papatasi* populations [74,75,76] and laboratory colonies [77], but induces only weak or insignificant CI [78,79]. The availability of cultures of both *w*Pip and *w*Pap in multiple different arthropod cell lines will facilitate the genome sequencing and phenotypic characterisation of these *Wolbachia* strains. The ability to propagate multiple different strains of *Wolbachia* in the same cell line will also allow the comparison of their properties, as previously proposed [4]. As well as the obvious difference that *w*Pip killed its parental host *C. pipiens* cells, whereas *w*Pap was tolerated by its parental host *P. papatasi* cells, we found differences in morphology, growth rate and habit and pathogenicity between *w*Pip (supergroup B) and *w*Pap (supergroup A) in the heterologous tick and insect cell lines. Intriguingly, in a preliminary experiment, infection with *w*Pap appeared to confer an advantage on IRE/CTVM20 tick cells, enabling them to grow to a higher density than uninfected cells. Further characterisation and comparison with other *Wolbachia* strains from the same and different supergroups grown in the cell lines used in the present study and other cell lines derived from the same genera, such as the *Culicoides sonorensis* KC cell line [80] and the *Ixodes ricinus* IRE11 cell line [24], will help to unravel the links between the various *Wolbachia* genomes and phenotypes and their effects on host cells. Notably, the *w*Stri strain, originally derived from the planthopper *Laodelphax striatellus* and maintained routinely in the *Ae. albopictus* cell line AeAl-2, was also found to be pathogenic to tick (ISE6) cells [24]. This *Wolbachia* strain was recently introduced stably into a heterologous planthopper, the destructive rice pest *Nilaparvata lugens*, in which the *w*Stri caused both CI and inhibition of rice ragged stunt virus [81].

Confirming previous reports [3,21,22], our study demonstrates how insect primary cultures can be successfully used to isolate *Wolbachia*, especially if the cultures are monitored from an early stage and the presence of bacteria is expected. Equally, proliferation of unanticipated and undetected *Wolbachia* could result in loss of primary cultures or young cell lines, as would probably have happened with CPE/LULS50 and CPL/LULS56 if the tetracycline treatment had not been initiated. Persistent infections with *Wolbachia* in insect cell lines may be unstable, as reported for the *Ae. albopictus* line Aa23 [82]; it will be interesting to determine how consistently *Wolbachia* persists in PPL/LULS49/*w*Pap cells at higher passage levels. As observed with insect cells, the presence, not always expected, of bacterial symbionts such as *Rickettsia* spp. and *Spiroplasma* spp. has resulted in either the loss of primary tick cell cultures but concomitant isolation of the microorganisms into heterologous tick cell lines [42,52], the persistence of bacteria in long-lived primary cultures [83] or a persistently-infected tick cell line [84].

Although ticks are not currently considered to be natural hosts of *Wolbachia* [85,86,87], our study confirmed the susceptibility of tick cells in vitro for multiple *Wolbachia* strains of differing insect origins [24]. As well as growing vigorously in all the tick cell lines tested, *w*Pip grew in insect cell lines derived from the lepidopteran *S. frugiperda*, the mosquito *Ae. albopictus*, the biting midge *C. nubeculosus* and the sand fly *L. longipalpis*. Other strains of *Wolbachia* have previously been propagated in the Sf9 and C6/36 cell lines used in the present study [4,19] and in other *L. longipalpis* and *Culicoides* sp. cell lines [80,88]. Interestingly, *w*Pap did not detectably infect the *C. nubeculosus* cell line CNE/LULS44, although it grew vigorously in all the other insect and tick cell lines tested. In all cases, infection with *Wolbachia* was achieved by simply adding a cell-free supernate to the recipient heterologous cultures, confirming previous results [24], without the need for centrifugation to increase contact between the bacteria and the host cells, as used in some other studies [4,19,25,80,88].

## 5. Conclusions

The new insect cell lines and cultured *Wolbachia* strains reported in the present study will facilitate research not only into interactions between the parent arthropods and the bacteria at the cellular level but, also, into human and veterinary pathogens carried by *C. pipiens*, *P. papatasi* and *L. longipalpis* and the influence of *Wolbachia* on their transmission [89,90].

## Figures and Tables

**Table 2:** Infectivity of the *Wolbachia* strains *w*Pip and *w*Pap for heterologous tick and insect cell lines.

Cell Line	*w*Pip from CPE/LULS50			*w*Pip from CPL/LULS56			*w*Pap from PPL/LULS49		
	Infected	CPE	Passaged	Infected	CPE	Passaged	Infected	CPE	Passaged
BME/CTVM23	+	+	+ ^1^	+	+	ND	+	+	+ ^1,2^
ISE6	+	+	ND	ND			+	+	ND
IRE/CTVM20	ND			+	+	ND	+	−	+ ^2^
Sf9	+	+/−	ND	ND			+	−	ND
C6/36	+	−	+ ^2^	ND			ND		
CNE/LULS44	ND			+	+	+ ^1^	−	−	−
LLE/LULS45	ND			+	+	+ ^1^	+	−	+ ^2^
LLL/LULS52	ND			+	+	ND	+	−	+ ^2^

^1^ Aliquot of the infected cell culture passaged onto naïve cells. ^2^ Infected culture split 1:1 at least once. Shaded cells indicate cell line/*Wolbachia* combination not tested; CPE = cytopathic effect seen; ND = not done; + = positive result; − = negative result.

## Data Availability

The novel gene sequences reported in this manuscript are deposited in GenBank under accession numbers MZ577346-MZ577349 and MZ577351-MZ577354.
